# Supplementary figures and images for: The phytoplasma SAP54 effector acts as a molecular matchmaker for leafhopper vectors by targeting plant MADS-box factor SVP
Source: eLife. 2025 Jan 7;13:RP98992. doi: 10.7554/eLife.98992 (PMC11706604; doi:10.7554/eLife.98992)

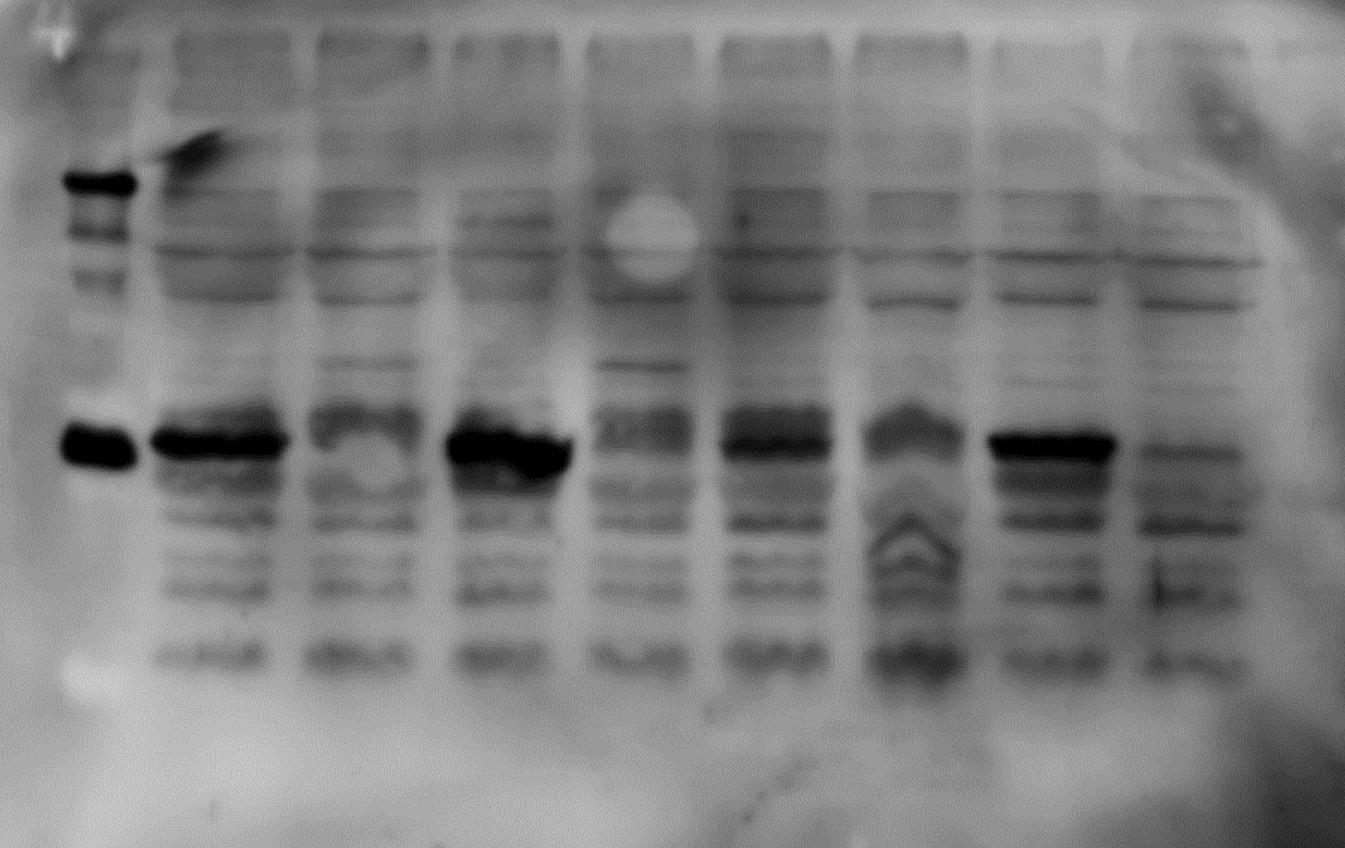

Supplement: Figure 5—figure supplement 1—source data 1. [file elife-98992-fig5-figsupp1-data1.zip › Figure 5-supplement1-source data1/Blot10_assay1.png]

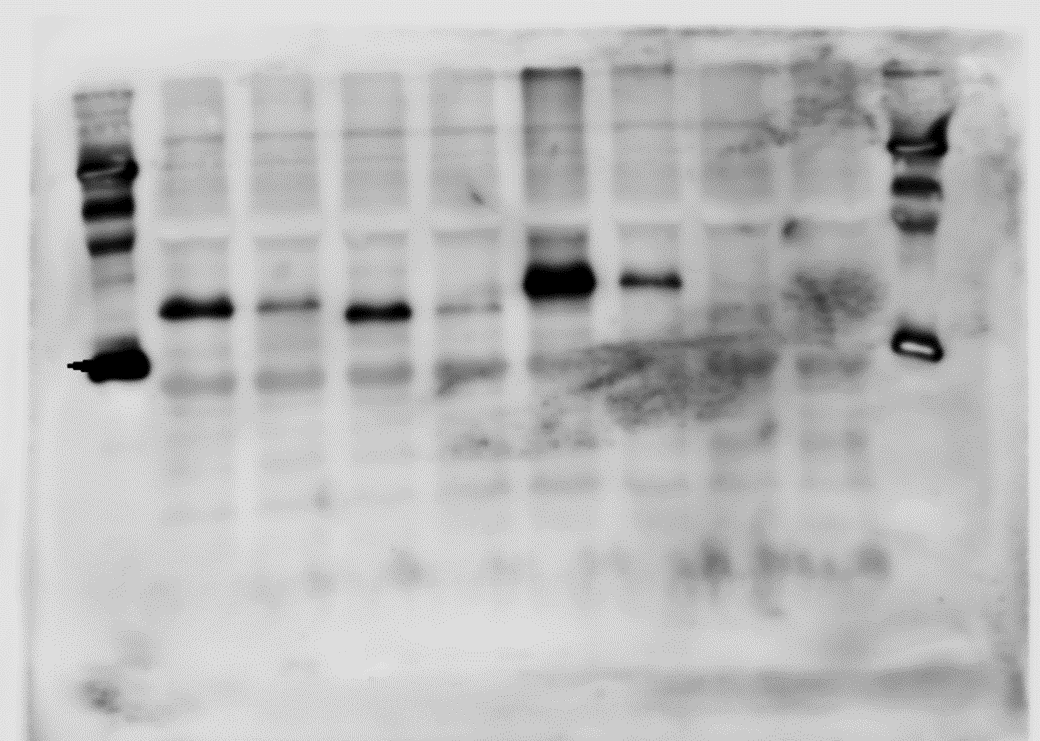

Supplement: Figure 5—figure supplement 1—source data 1. [file elife-98992-fig5-figsupp1-data1.zip › Figure 5-supplement1-source data1/Blot1_assay2.png]

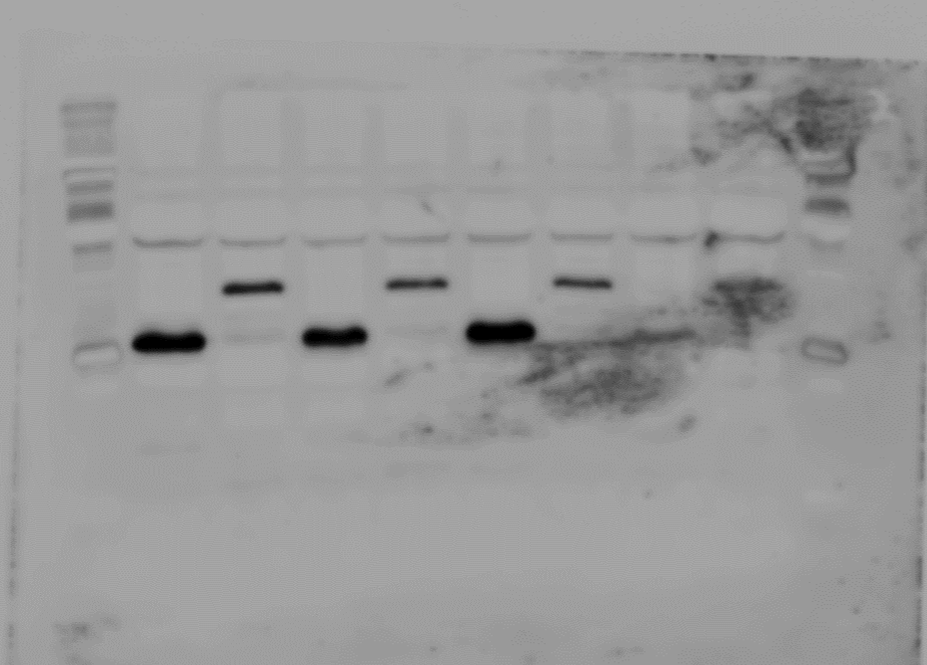

Supplement: Figure 5—figure supplement 1—source data 1. [file elife-98992-fig5-figsupp1-data1.zip › Figure 5-supplement1-source data1/Blot2_assay2.png]

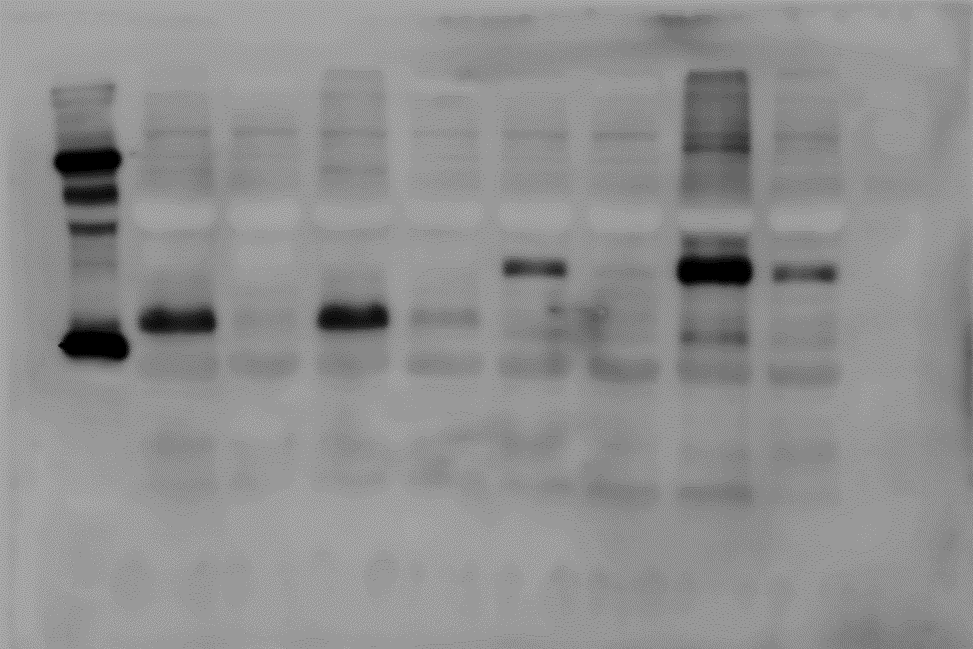

Supplement: Figure 5—figure supplement 1—source data 1. [file elife-98992-fig5-figsupp1-data1.zip › Figure 5-supplement1-source data1/Blot3_assay2.png]

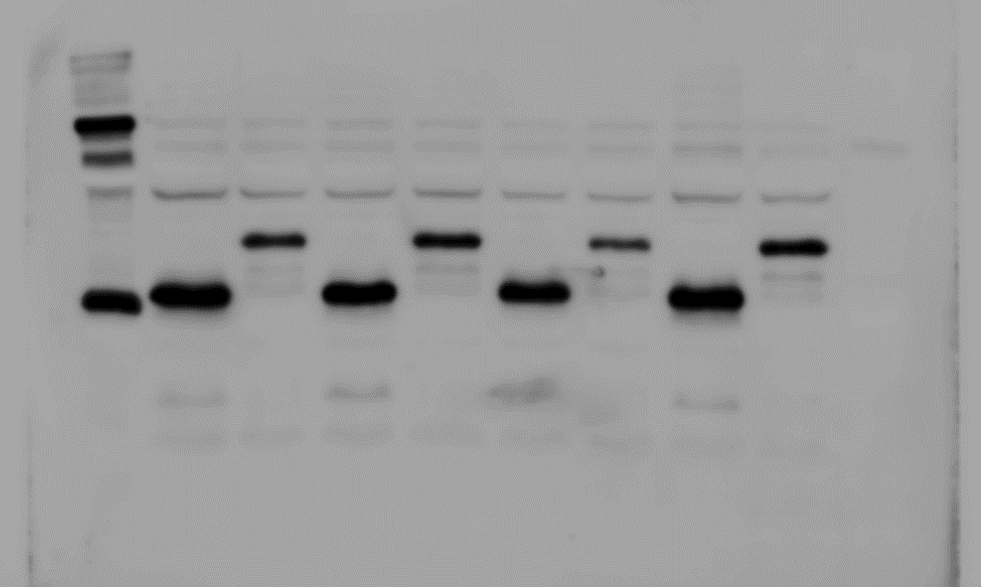

Supplement: Figure 5—figure supplement 1—source data 1. [file elife-98992-fig5-figsupp1-data1.zip › Figure 5-supplement1-source data1/Blot4_assay2.png]

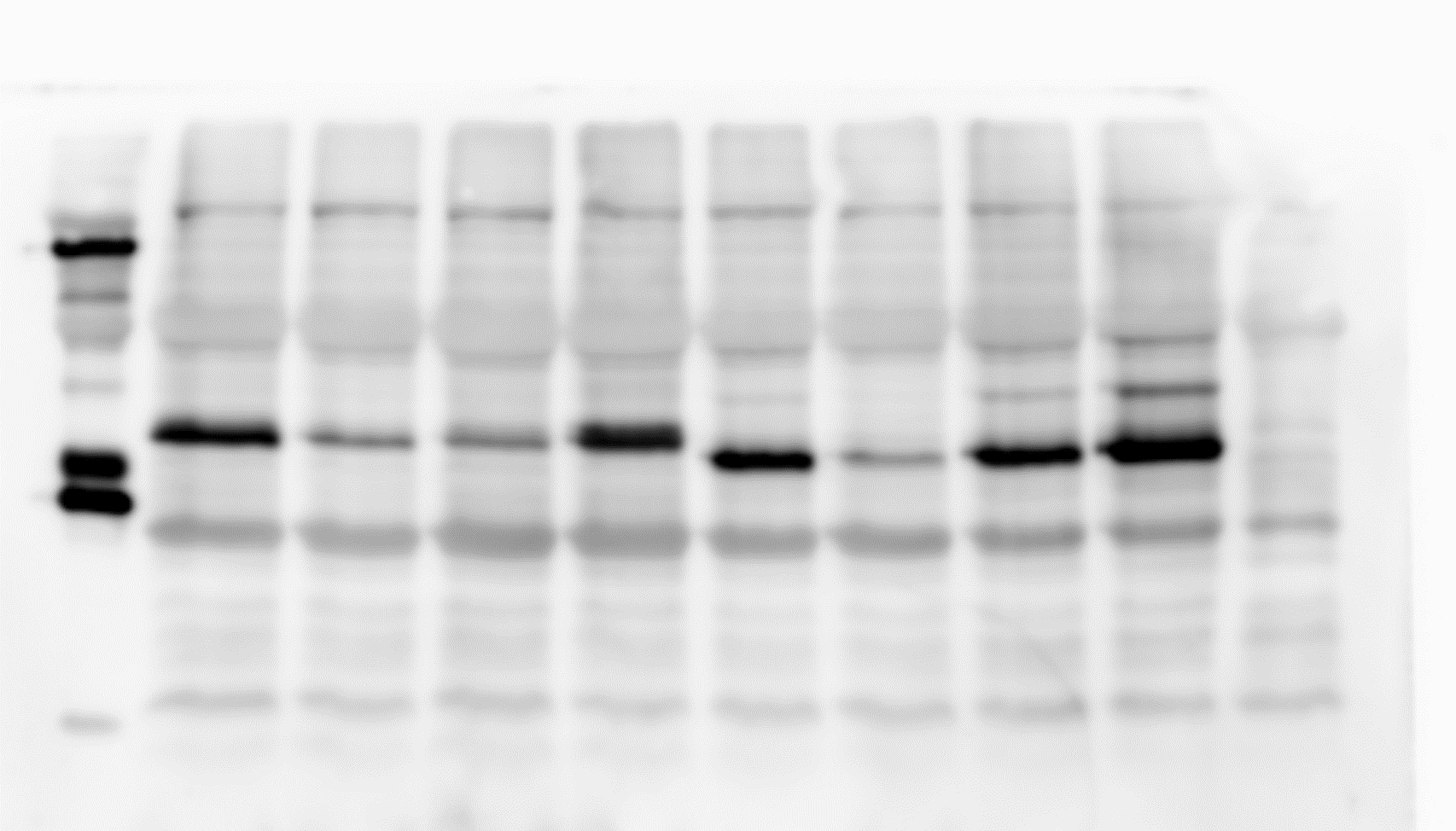

Supplement: Figure 5—figure supplement 1—source data 1. [file elife-98992-fig5-figsupp1-data1.zip › Figure 5-supplement1-source data1/Blot5_assay1.png]

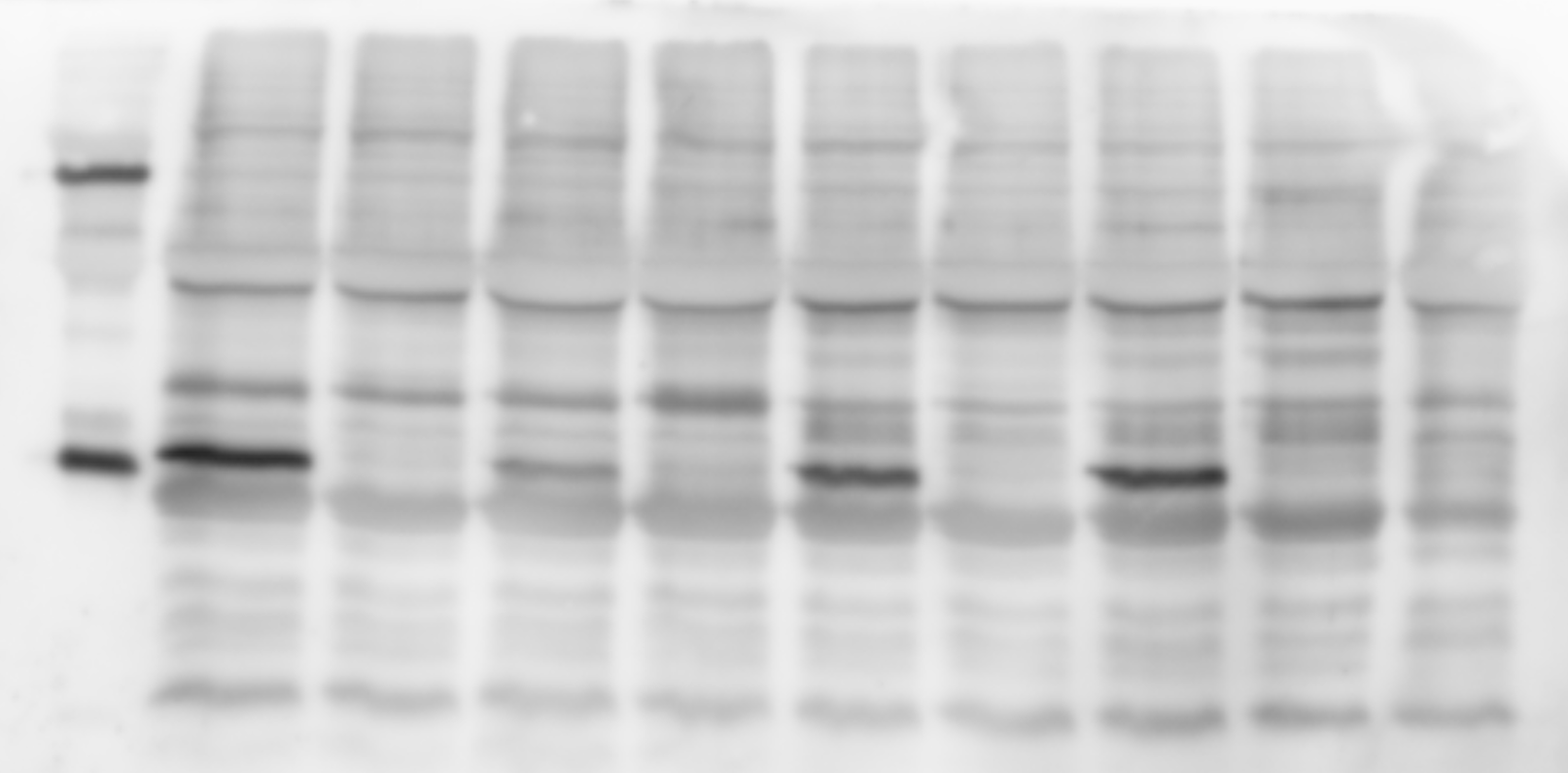

Supplement: Figure 5—figure supplement 1—source data 1. [file elife-98992-fig5-figsupp1-data1.zip › Figure 5-supplement1-source data1/Blot6_assay1.png]

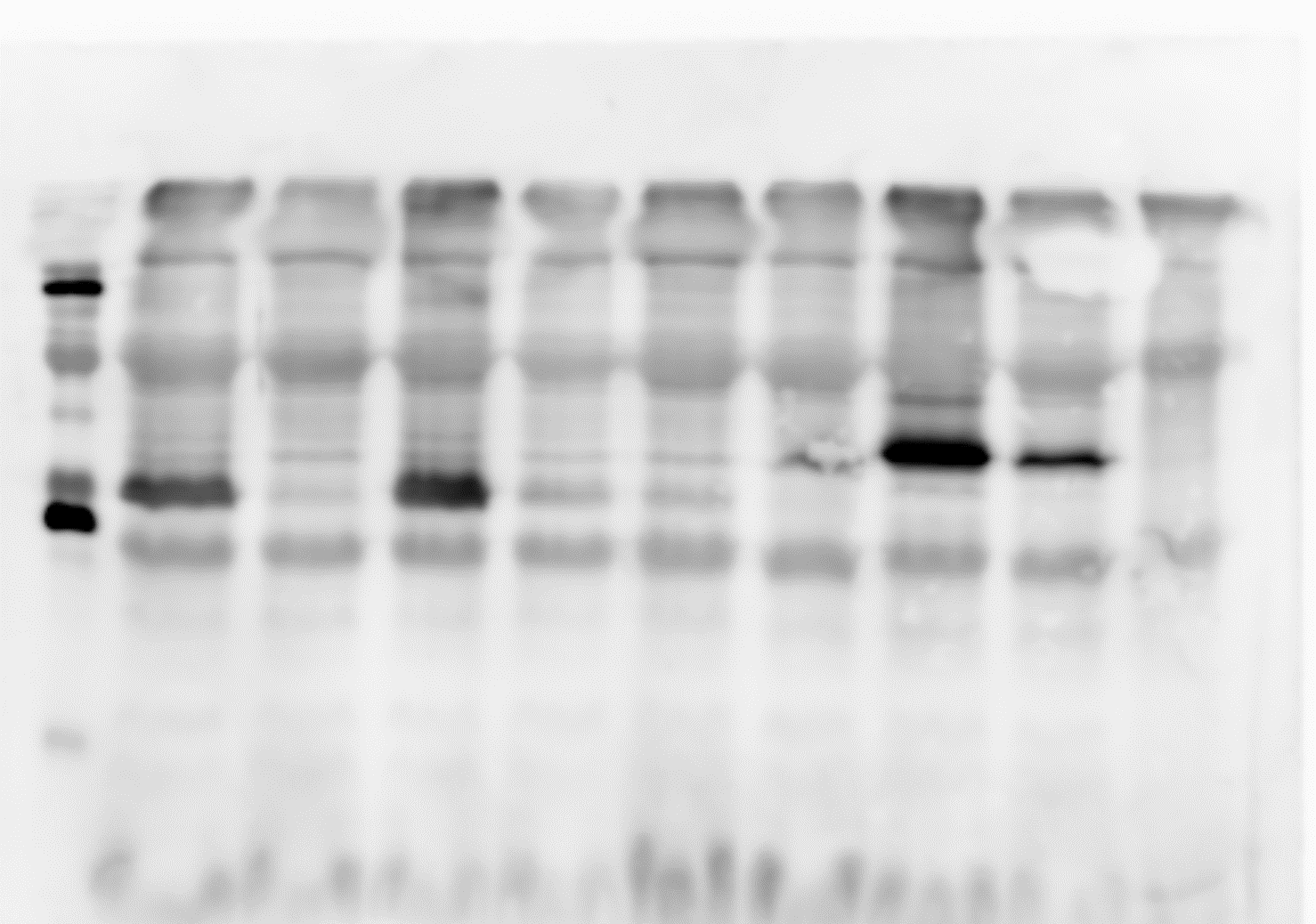

Supplement: Figure 5—figure supplement 1—source data 1. [file elife-98992-fig5-figsupp1-data1.zip › Figure 5-supplement1-source data1/Blot7_assay1.png]

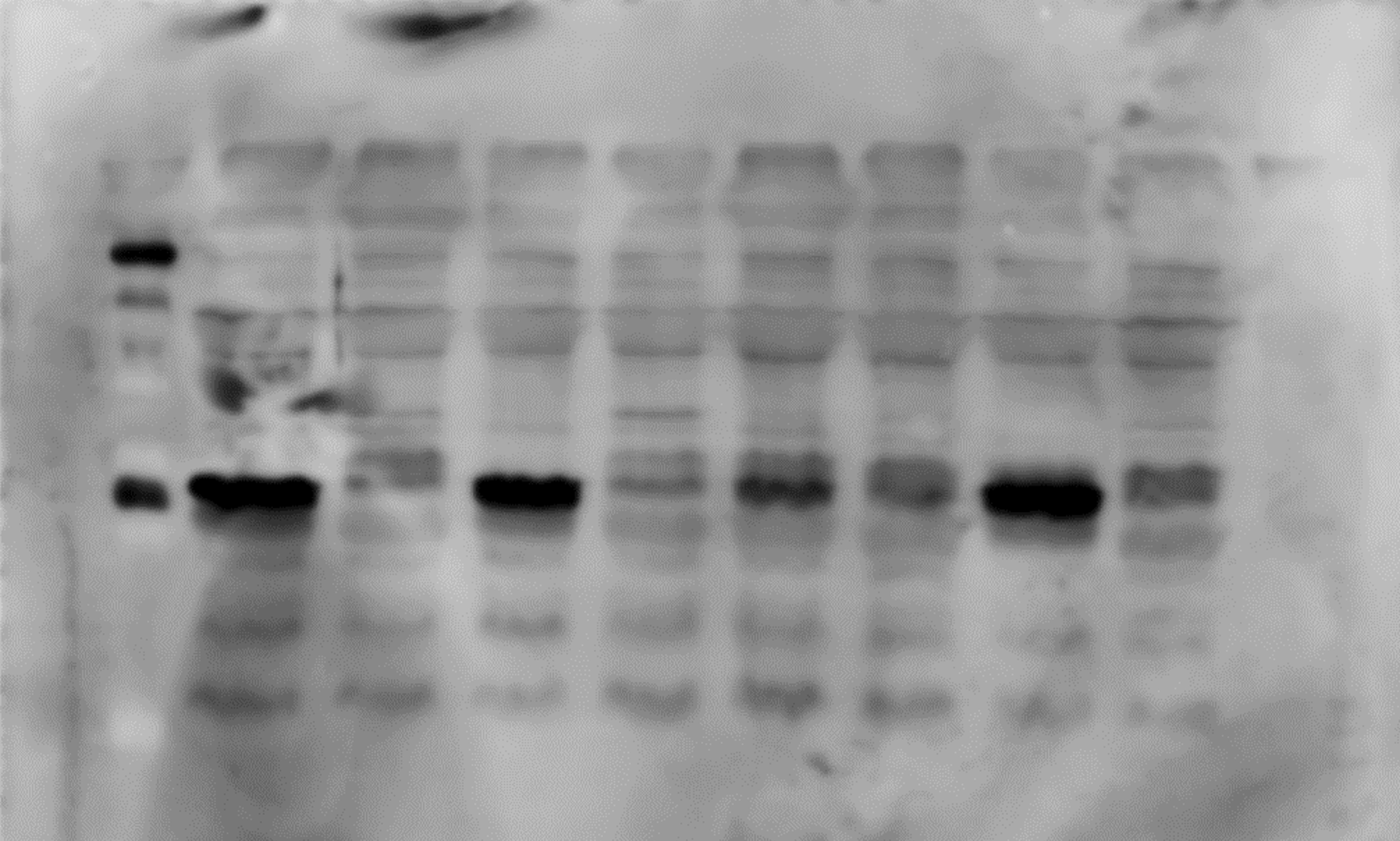

Supplement: Figure 5—figure supplement 1—source data 1. [file elife-98992-fig5-figsupp1-data1.zip › Figure 5-supplement1-source data1/Blot8_assay1.png]

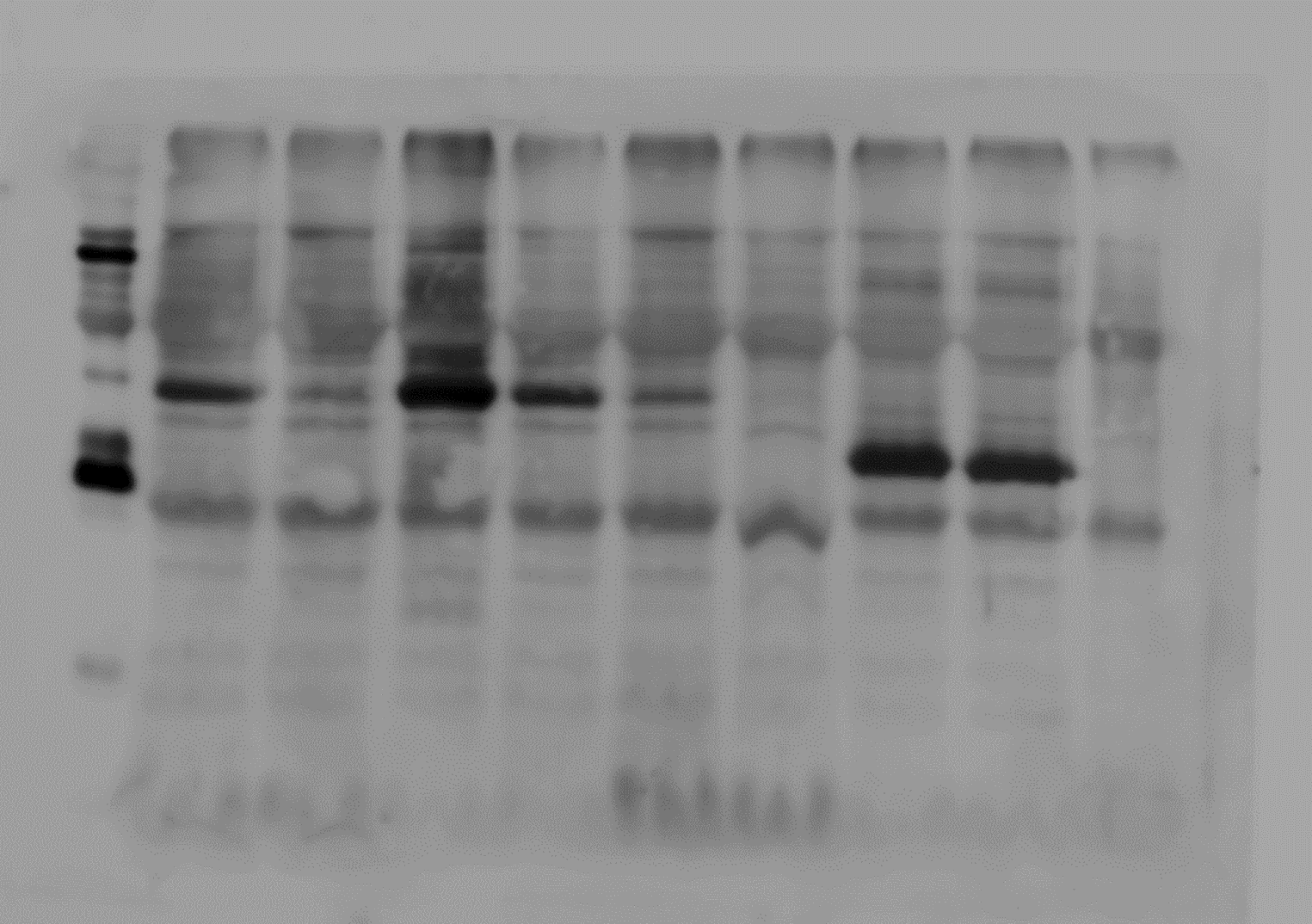

Supplement: Figure 5—figure supplement 1—source data 1. [file elife-98992-fig5-figsupp1-data1.zip › Figure 5-supplement1-source data1/Blot9_assay1.png]

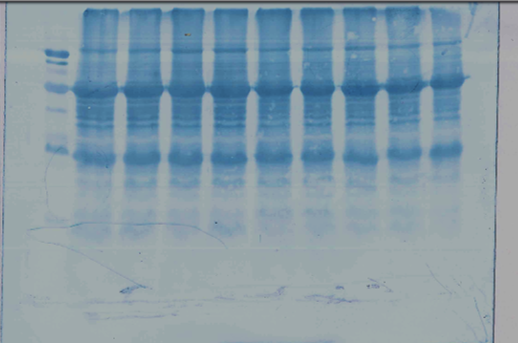

Supplement: Figure 5—figure supplement 1—source data 1. [file elife-98992-fig5-figsupp1-data1.zip › Figure 5-supplement1-source data1/Coumassie1_assay2.png]

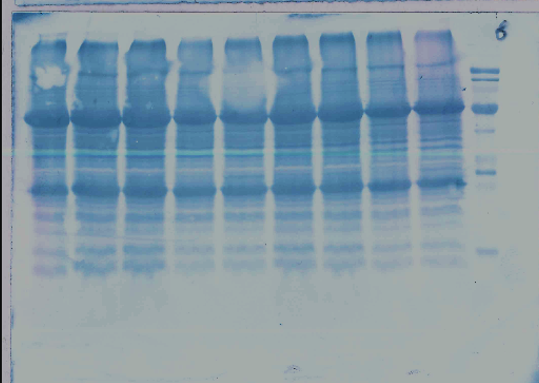

Supplement: Figure 5—figure supplement 1—source data 1. [file elife-98992-fig5-figsupp1-data1.zip › Figure 5-supplement1-source data1/Coumassie2_assay2.png]

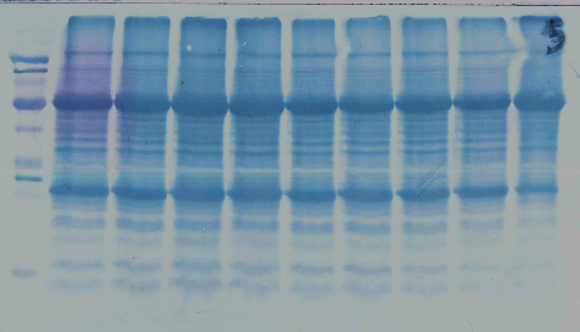

Supplement: Figure 5—figure supplement 1—source data 1. [file elife-98992-fig5-figsupp1-data1.zip › Figure 5-supplement1-source data1/Coumassie3_assay1.png]

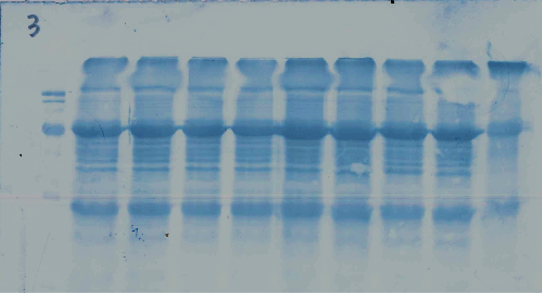

Supplement: Figure 5—figure supplement 1—source data 1. [file elife-98992-fig5-figsupp1-data1.zip › Figure 5-supplement1-source data1/Coumassie4_assay1.png]

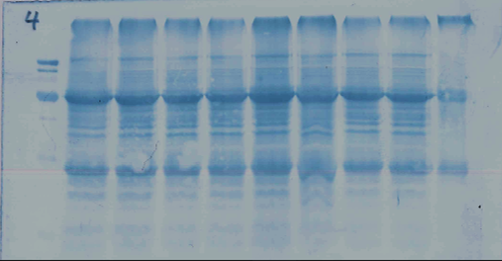

Supplement: Figure 5—figure supplement 1—source data 1. [file elife-98992-fig5-figsupp1-data1.zip › Figure 5-supplement1-source data1/Coumassie5_assay1.png]

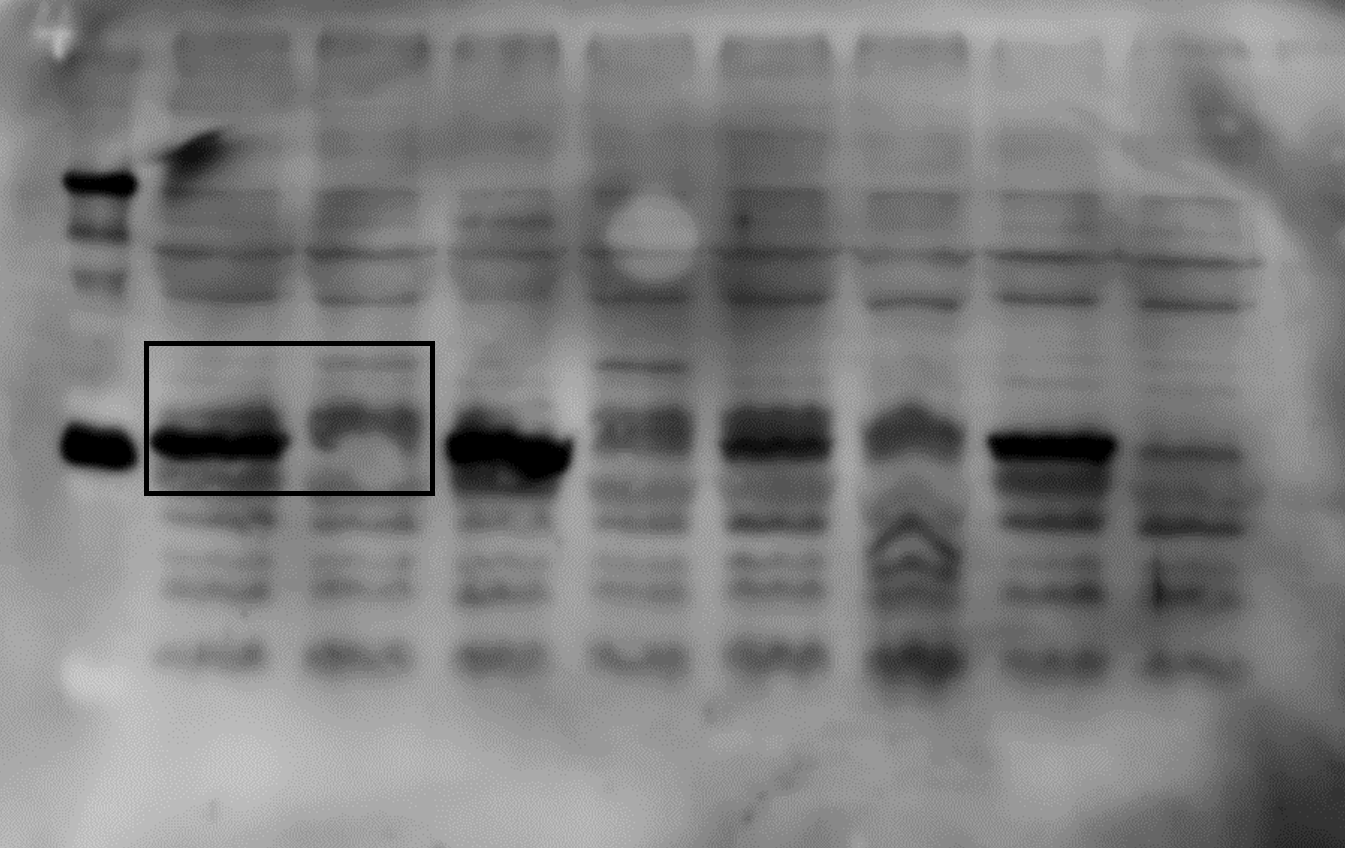

Supplement: Figure 5—figure supplement 1—source data 2. [file elife-98992-fig5-figsupp1-data2.zip › Figure 5-supplement1-source data2/Blot10_assay1.png]

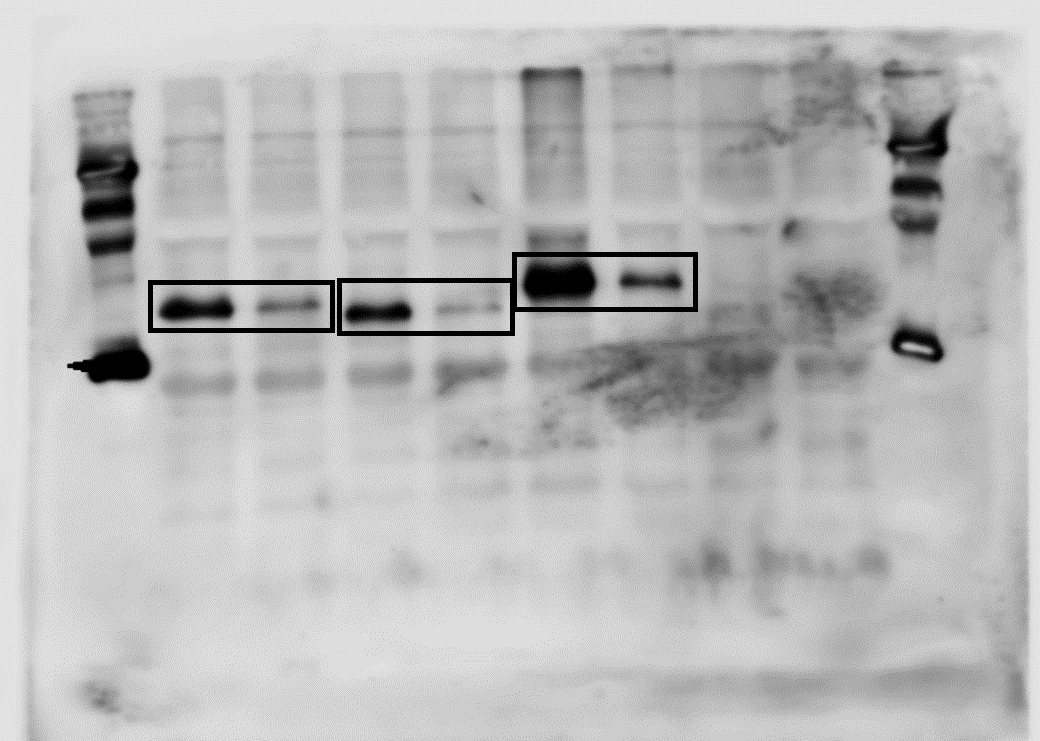

Supplement: Figure 5—figure supplement 1—source data 2. [file elife-98992-fig5-figsupp1-data2.zip › Figure 5-supplement1-source data2/Blot1_assay2.png]

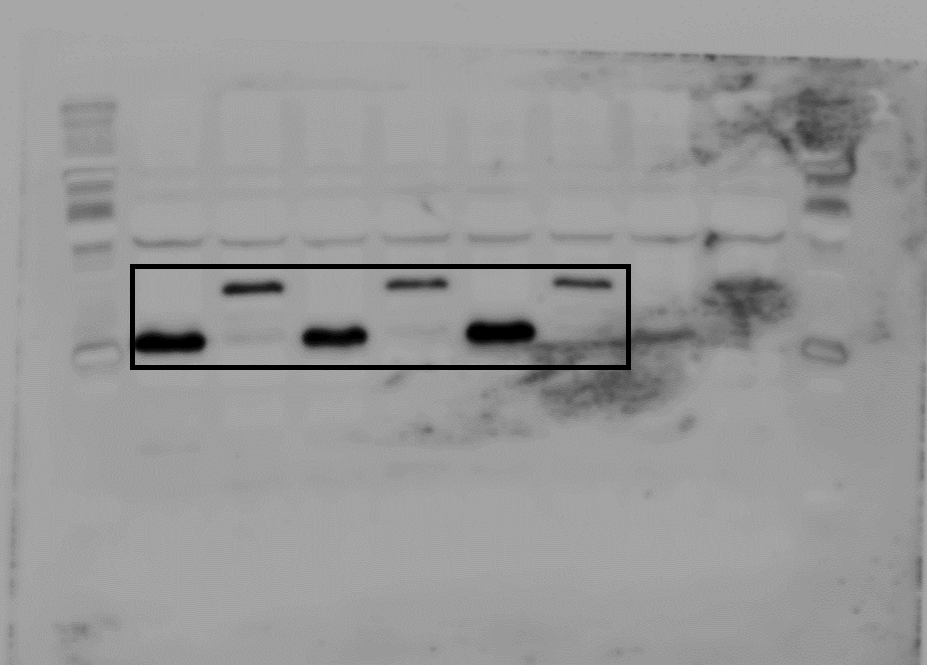

Supplement: Figure 5—figure supplement 1—source data 2. [file elife-98992-fig5-figsupp1-data2.zip › Figure 5-supplement1-source data2/Blot2_assay2.png]

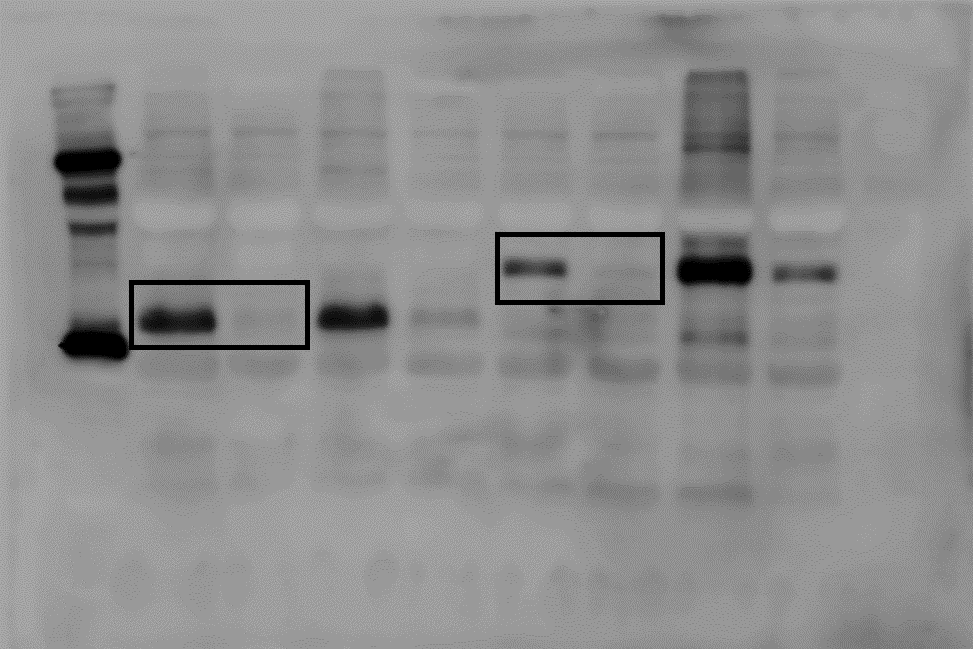

Supplement: Figure 5—figure supplement 1—source data 2. [file elife-98992-fig5-figsupp1-data2.zip › Figure 5-supplement1-source data2/Blot3_assay2.png]

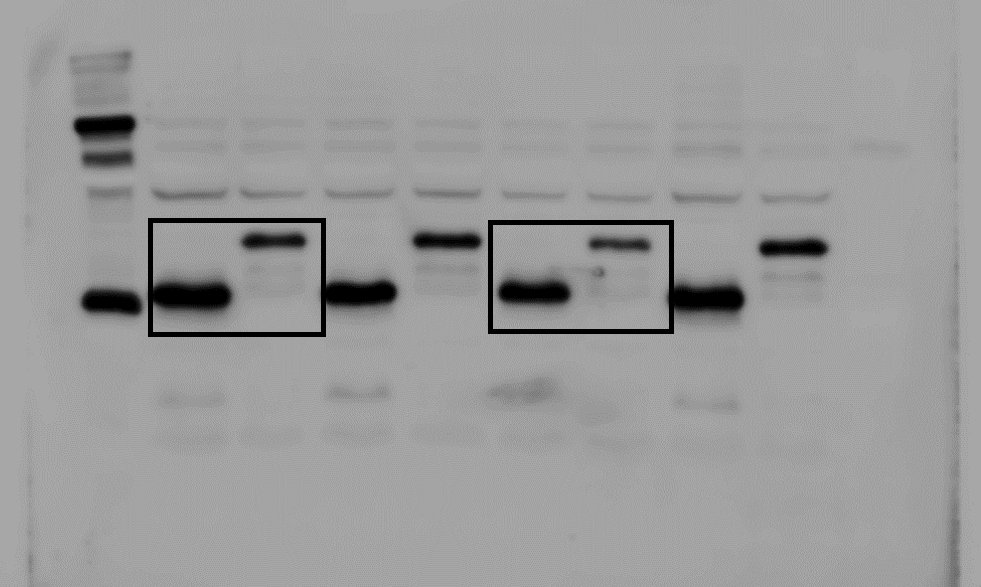

Supplement: Figure 5—figure supplement 1—source data 2. [file elife-98992-fig5-figsupp1-data2.zip › Figure 5-supplement1-source data2/Blot4_assay2.png]

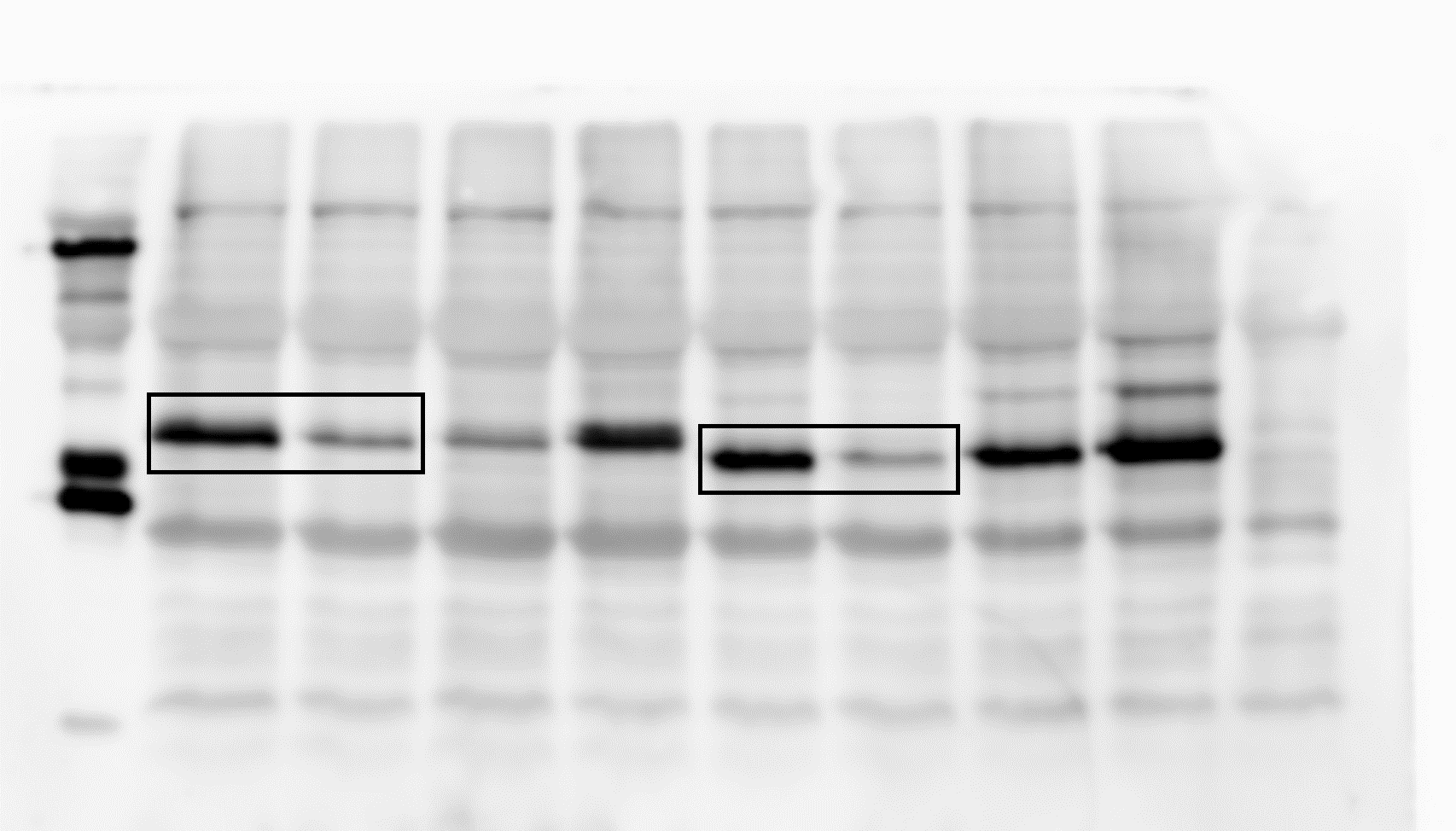

Supplement: Figure 5—figure supplement 1—source data 2. [file elife-98992-fig5-figsupp1-data2.zip › Figure 5-supplement1-source data2/Blot5_assay1.png]

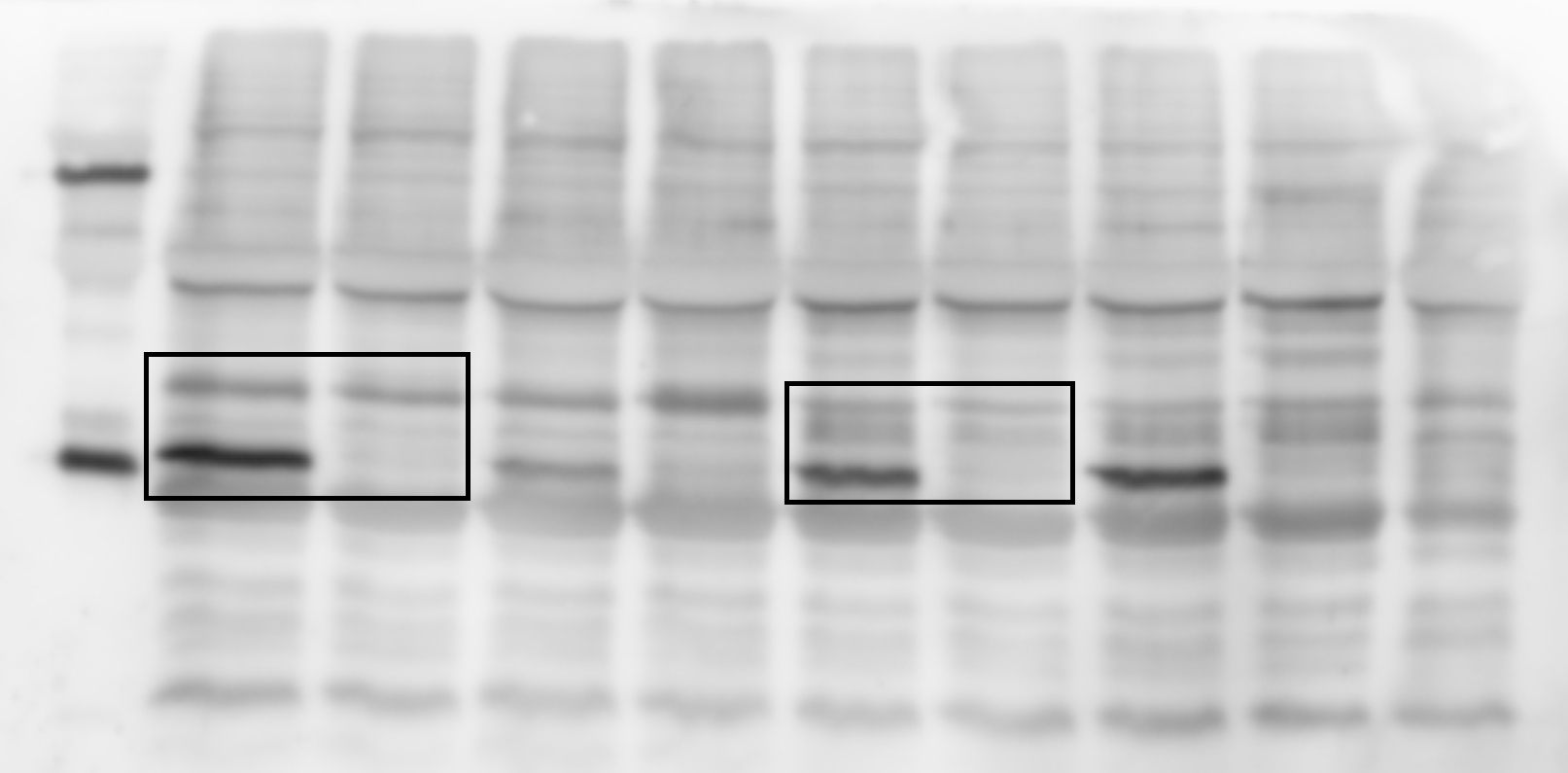

Supplement: Figure 5—figure supplement 1—source data 2. [file elife-98992-fig5-figsupp1-data2.zip › Figure 5-supplement1-source data2/Blot6_assay1.png]

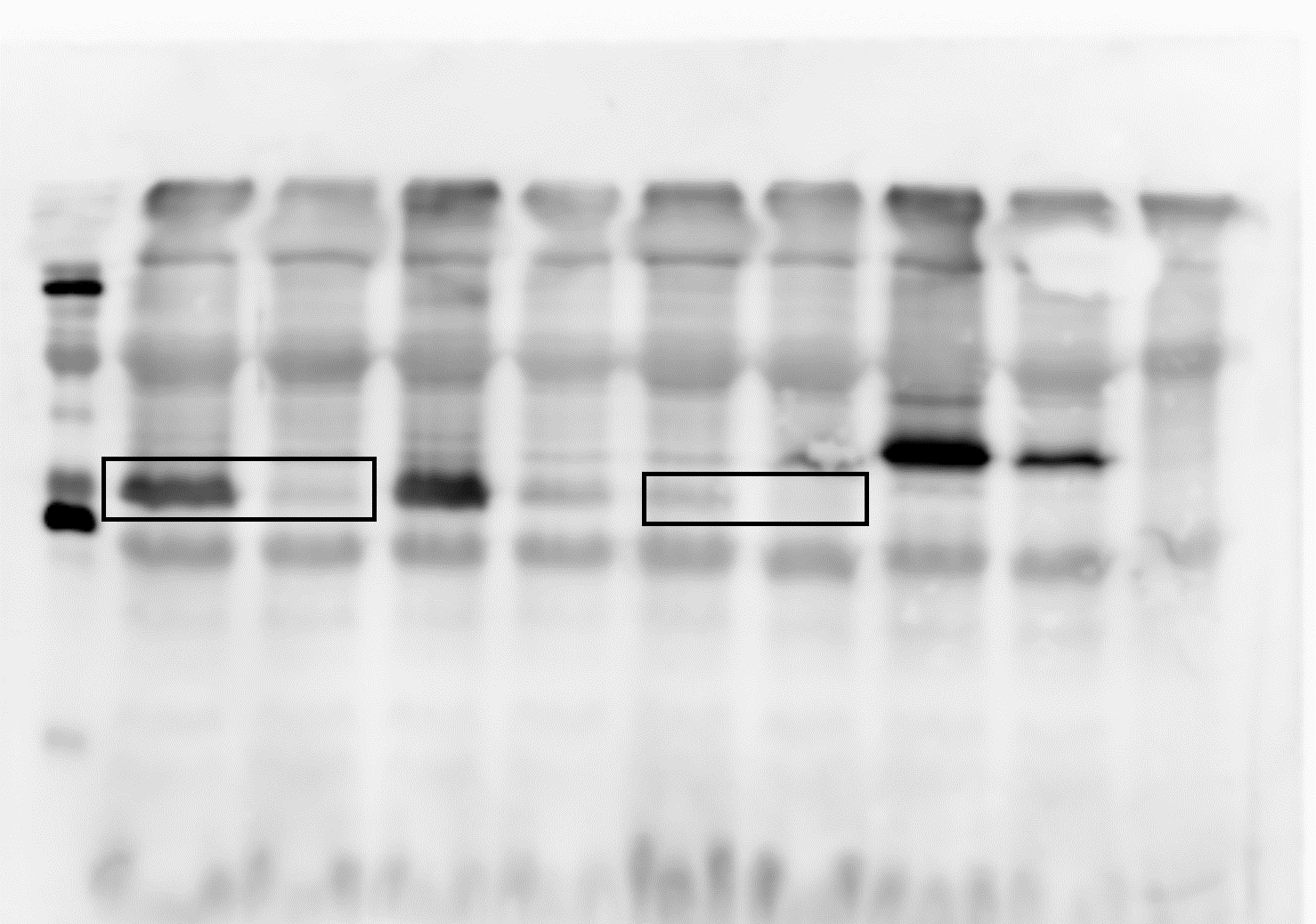

Supplement: Figure 5—figure supplement 1—source data 2. [file elife-98992-fig5-figsupp1-data2.zip › Figure 5-supplement1-source data2/Blot7_assay1.png]

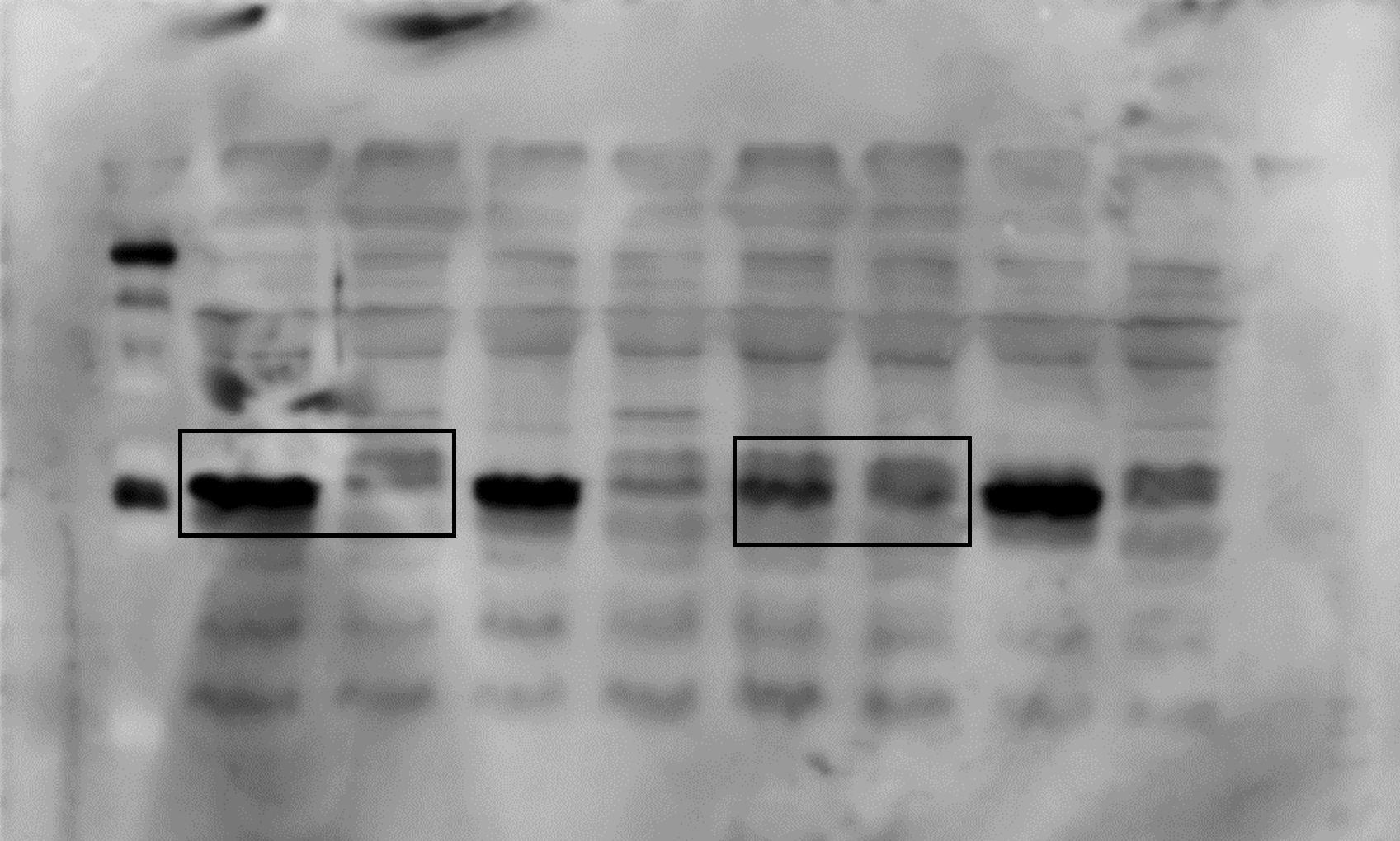

Supplement: Figure 5—figure supplement 1—source data 2. [file elife-98992-fig5-figsupp1-data2.zip › Figure 5-supplement1-source data2/Blot8_assay1.png]

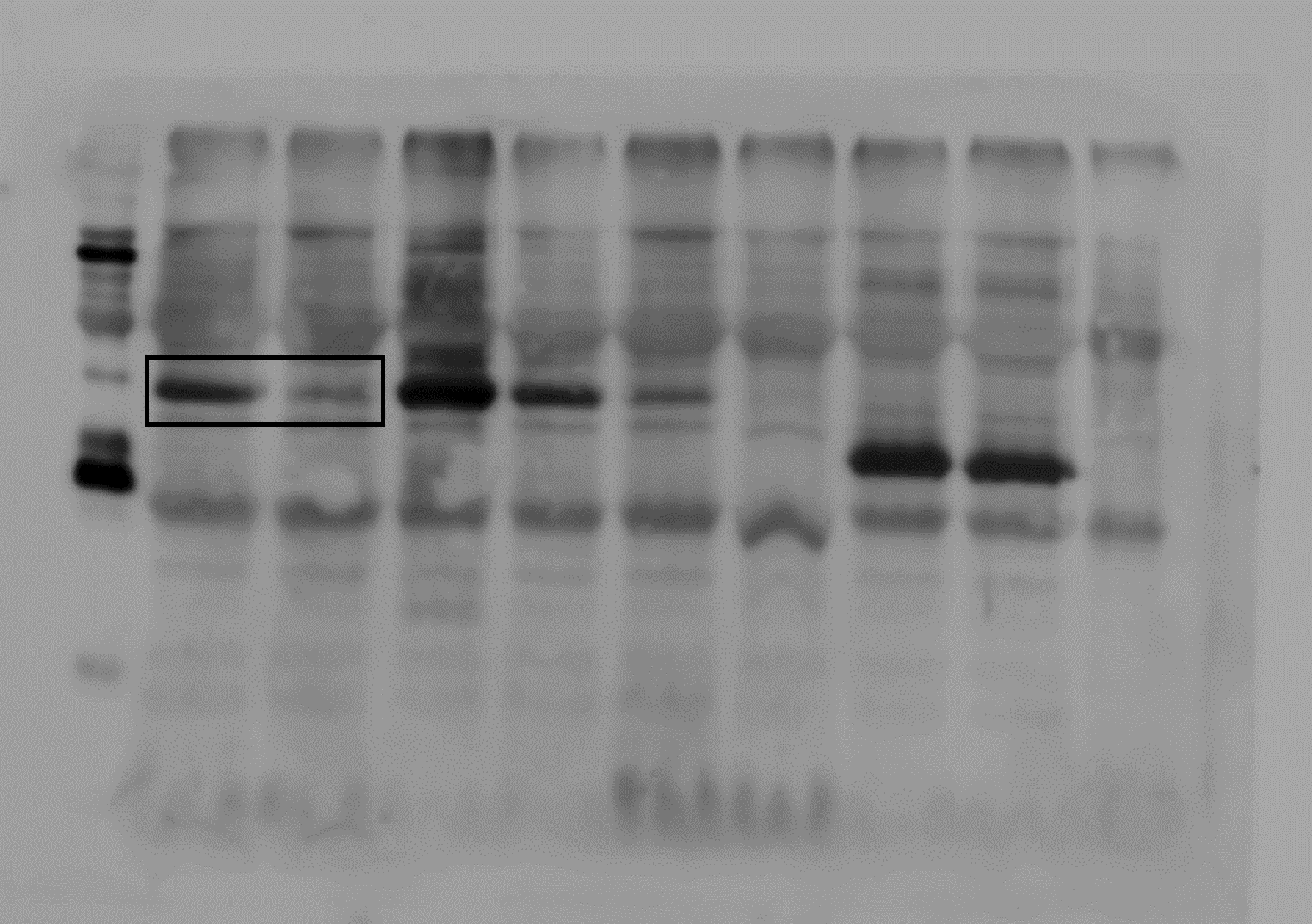

Supplement: Figure 5—figure supplement 1—source data 2. [file elife-98992-fig5-figsupp1-data2.zip › Figure 5-supplement1-source data2/Blot9_assay1.png]

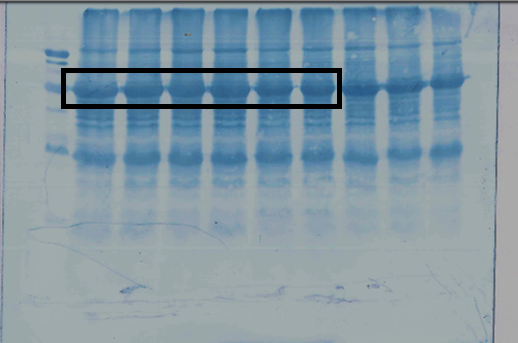

Supplement: Figure 5—figure supplement 1—source data 2. [file elife-98992-fig5-figsupp1-data2.zip › Figure 5-supplement1-source data2/Coumassie1_assay2.png]

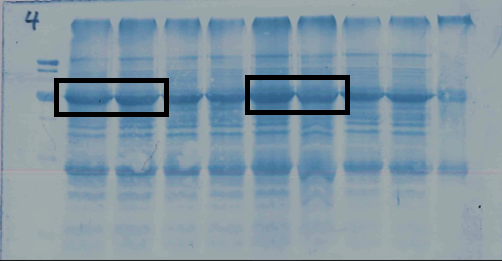

Supplement: Figure 5—figure supplement 1—source data 2. [file elife-98992-fig5-figsupp1-data2.zip › Figure 5-supplement1-source data2/Coumassie2_assay2.png]

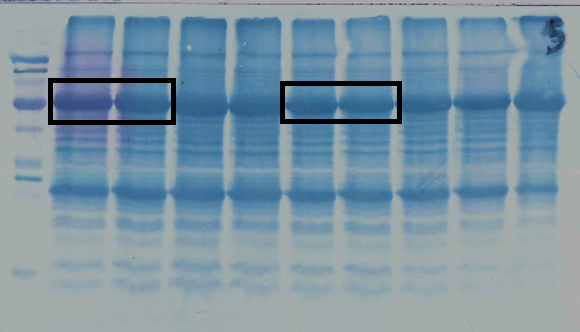

Supplement: Figure 5—figure supplement 1—source data 2. [file elife-98992-fig5-figsupp1-data2.zip › Figure 5-supplement1-source data2/Coumassie3_assay1.png]

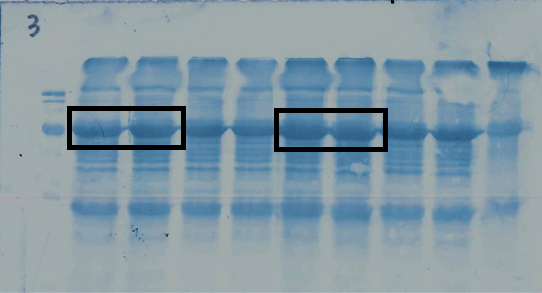

Supplement: Figure 5—figure supplement 1—source data 2. [file elife-98992-fig5-figsupp1-data2.zip › Figure 5-supplement1-source data2/Coumassie4_assay1.png]

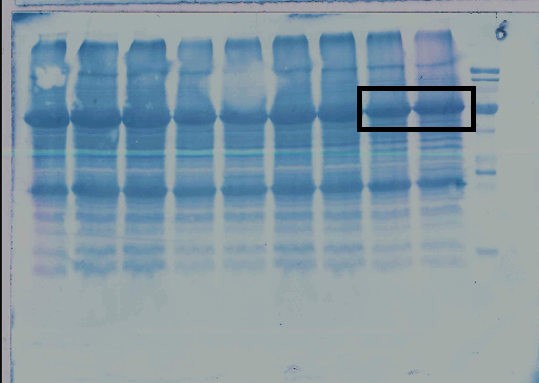

Supplement: Figure 5—figure supplement 1—source data 2. [file elife-98992-fig5-figsupp1-data2.zip › Figure 5-supplement1-source data2/Coumassie5_assay1.png]
